# Minimally invasive techniques in the management of muscular temporomandibular joint disorders: A five-year observational study

**DOI:** 10.4317/jced.61618

**Published:** 2024-05-01

**Authors:** Mario-Roberto Homem, Debora-Lemos Backer, Franciele Floriani, Carlos A. Jurado, Kelvin I. Afrashtehfar

**Affiliations:** 1 DDS, MSc. Clinical Professor, Department of Prosthodontics, Brazilian Association of Dentistry, UniABOSC; 2 DDS. Private Practice, Brasilia, Brazil; 3 DDS, MSc, PhD. Clinical Assistant Professor, Department of Prosthodontics, The University of Iowa College of Dentistry and Dental Clinics, Iowa City, Iowa, USA; 4 Associate Professor and Director of Operative Dentistry Division, Division of Operative Dentistry, Department of General Dentistry, The University of Tennessee Health Science Center; 5 DDS, MSc, PhD, FDS RCS, FRCDC. Assistant Professor and Director of Evidence-Based Practice Unit, Clinical Sciences. Department, College of Dentistry, Ajman University, Ajman City, AE, United Arab Emirates; 6 DDS, MSc, PhD, FDS RCS, FRCDC. Department of Reconstructive Dentistry and Gerodontology, School of Dental Medicine, University of Bern, Bern, BE, Switzerland

## Abstract

**Background:**

To evaluate the effectiveness of three minimally invasive techniques for managing patients with myofascial pain dysfunction, determine their association with sociodemographic factors, habits, medication usage, comorbidities, treatment history, pain duration, complaint intensity, and diagnosis limitations.

**Material and Methods:**

This five-year observational study scrutinized 1,000 medical records from individuals treated at the TMD Orofacial Dental Research Center. TMD treatments were organized into Group 1 (thermotherapy, exercises, and CBT), Group 2 (Group 1 plus intramuscular manual therapy), and Group 3 (Group 1 and Group 2 plus occlusal appliances) and correlated with sociodemographic factors, habits, prior medication usage, comorbidities, history of prior treatments, duration of pain, intensity of complaint, and diagnosis limitations or without limitations regarding the symptoms of muscular temporomandibular disorders (TMD).

**Results:**

Treatment durability was proportionally higher in Groups II and III (*p*<0.05). Although no significant differences were found for habits (*p*= 0.051) and pain duration (*p*= 0.001), clenching was more prevalent in Groups II n= 77 (57.0%) and III n= 39 (63.9%) and among those with therapy duration equal to or greater than 6 months for n=102 (59.3%). Statistically significant correlations were noted between age and education (rho=-0.198; *p*<0.001) and between pain duration and treatment durability (rho=0.317; *p*<0.001).

**Conclusions:**

Intraoral devices do not constitute the primary treatment for myofascial pain. For cases of prolonged pain, comorbidities, limited mouth opening, and a history of prior medication or treatments, a splint combined with other therapies is recommended for effective management.

** Key words:**Temporomandibular disorders, myofascial pain, occlusal appliances, clinical diagnosis, thermotherapy, exercise therapy, cognitive behavioral therapy.

## Introduction

Temporomandibular disorders (TMD) represent a diverse spectrum of multifactorial conditions affecting the temporomandibular joint (TMJ), muscles, articulation, and facial nerves, characterized by functional alterations in the masticatory apparatus (1). Identifying a single triggering etiological factor is challenging due to their multifactorial origin, arising from a complex interplay of psychological, structural, and postural factors that can disrupt the masticatory muscles and temporomandibular joint (2,3). Psychological conditions, often associated with tension leading to bruxism (teeth grinding and clenching), have been linked to the development of TMD (4,5).

The literature highlights a diverse range of treatments for temporomandibular disorders with muscular origin, involving individualized combinations of therapies. These options include counseling, physiotherapy, jaw exercises, pharmacologic interventions, behavioral medicine, and physical therapies such as acupuncture, dry needling, transcutaneous electrical nerve stimulation (TENS), and the use of heat and cold applications, along with occlusal appliances (6-8).

Occlusal appliances have traditionally been a common treatment for painful TMDs, yet the evidence base for their efficacy remains unclear and subject to questioning (9). Potential mechanisms include alterations in the reflective pattern of the masticatory muscles, reduction in loading on the masticatory muscles and TMJs, heightened awareness of parafunctional activity, or a placebo effect (10-13). Previous studies combining occlusal splint treatment with other modalities have demonstrated impressive results in clinical symptoms, reflecting the complexity of TMD management (15,16). A systematic review, assessing the efficacy of occlusal appliances in managing painful TMDs, explored the role of the placebo effect. Contrary to expectations, patient-reported treatment satisfaction extended beyond pain intensity, including improvements in physical functioning and psychosocial factors, suggesting a treatment effect beyond placebo (17). Lastly, this five-year observational study aims to compare minimally invasive techniques for managing myofascial pain in patients, considering the presence or absence of opening mouth limitations.

## Material and Methods

-Design and patient data collection

Observational study based on an analysis of clinical patient records was performed at the Temporomandibular Dysfunction and Orofacial Pain Clinic of the Dental Research Center between May 2015 and March 2020. A pilot study determined the sample size (n) with an estimated target population of 480. Ensuring reliable statistical analyses, a sample size of 348 files was calculated based on the Central Limit Theorem and the Laws of Large Numbers, assuming a 3.0% error rate.

The anonymized patient data had the following patient inclusion criteria.

• Over 18 years of age, of both genders

• Diagnosed with myofascial pain with or without limitation of mouth opening (Ia and Ib) according to the Research Diagnostic Criteria for Temporomandibular Disorder (RDC/TMD) Axis I (18).

Whereas the exclusion criterion was clinical files with incomplete data.

-Evaluated minimally invasive techniques

The TMD treatment techniques were organized into three groups:

• Group 1: Thermotherapy, therapeutic exercises, and cognitive-behavioral therapy (CBT).

• Group 2: Therapies from Group 1 and intramuscular manual therapy (dry needling).

• Group 3: Therapies from Group 1, Group 2, and occlusal appliances.

These therapies in Groups I, II, and III were correlated with sociodemographic factors, habits (clenching, oncophagia, biting lips, posture), prior medication usage (yes or no), comorbidities (absent or exist), history of prior treatments (yes or no), duration of pain (up to 6 months, 6 months or more), intensity of complaint (light, moderate, or more), and diagnosis with clinical limitations or without limitations regarding TMD symptoms (myofascial pain, myofascial pain with limited opening, disc displacement with reduction, and arthragy).

-Statistical analysis

Data were presented using absolute (n) and relative frequency (%). Chi-square tests were employed for comparisons based on therapy type and treatment time. Spearman correlation coefficients assessed relationships between age, education, duration of initial complaint, and treatment time durability.

Crude and adjusted multinomial logistic regression models examined associations of sociodemographic, behavioral, and health-related characteristics with therapy type, using Therapy I as the reference. The backward model (Wald) excluded variables with *p* > 0.10 for adjusted analysis.

The association of sociodemographic, behavioral, and health-related characteristics with treatment duration was assessed using crude and adjusted binary logistic regression models. Treatment durability up to 6 months was the reference category. The backward model (Wald) excluded variables with *p* > 0.10. All analyses were conducted using SPSS Statistics for Windows, version 26.0, with a significance level of *p* < 0.05.

## Results

Study population and sociodemographic characteristics

In adherence to inclusion criteria, 348 dental records were analyzed from a total of 1,000, with exclusions attributed to insufficient data. A detailed descriptive analysis explored the correlation between behavioral characteristics, patients’ medical history, and treatment outcomes.

No statistically significant differences were observed in sociodemographic characteristics based on therapy type and treatment durability (*p*>0.05). The majority of participants were female, aged 40 or older, possessing a graduate degree, and engaged in some form of job occupation (part-time or full-day) ( Table 1).

 Table 2 displayed statistically significant differences in prior medication, comorbidity, prior treatment, intensity of complaint, and diagnosis limitations based on therapy type. Treatment durability was proportionally higher in Groups II and III (*p* < 0.05). Although no significant differences were found for habits (*p*= 0.051) and pain duration (*p*= 0.001), clenching was more prevalent in Groups II n= 77 (57.0%) and III n= 39 (63.9%) and among those with therapy duration equal to or greater than 6 months for n=102 (59.3%). Statistically significant correlations were noted between age and education (rho=-0.198; *p*<0.001) and between pain duration and treatment durability (rho=0.317; *p*<0.001) ( Table 3).

-Characteristics associated with therapy type

 Table 4 presented crude and adjusted characteristics associated with therapy type, using therapy I as the reference. In the adjusted analysis, education, habits, prior medication, comorbidity, prior treatment, and diagnosis remained in the final model. Variables like prior medication (OR=3.55; 95%CI=1.99-6.32; *p*<0.001), prior treatment (OR=2.62; 95%CI=1.41-4.87; *p*=0.002), and clinical diagnosis with limitations (OR=2.85; 95%CI=1.56-5.21; *p*=0.001) were associated with a higher chance of being in therapy group II compared to therapy group I. Additionally, variables such as clenching as a habit (OR=2.38; 95%CI=1.18-4.81; *p*=0.016), prior medication (OR=3.74; 95 %CI=1.59-8.81; *p*=0.003), present comorbidity (OR=3.16; 95%IC=1.52-6.58; *p*=0.002), and prior treatment (OR=6.36; 95%CI=3.05-13.27; *p*<0.001) were associated with a higher chance of being in therapy group III compared to therapy group I.

-Characteristics associated with therapy duration

 Table 5 presented crude and adjusted analyses of participant characteristics associated with therapy duration. In the adjusted analysis, habits, comorbidity, duration of pain, and clinical diagnosis variables remained in the final model. Variables like comorbidity (OR=2.31; 95%CI=1.42-3.77; *p*=0.001), pain duration of 6 months or more (OR=2.24; 95%CI=1.39-3.60; *p*=0.001), and clinical diagnosis with limitations (OR=2.02; 95%CI=1.21-3.38; *p*=0.007) were associated with a higher chance of needing 6 months or more of treatment compared to their peers.

-Treatment durability analysis by therapy type

 Table 6 provided a detailed analysis of treatment durability for the three therapies adopted. In Group I, the average treatment duration was 5.4 months, ranging from 2 to 12 months, with 50% completing treatment within a maximum of 5 months. In Group II, the average treatment duration was 7.5 months, ranging from 2 to 23 months, with 50% completing treatment within a maximum of 7 months. In Group III, the average treatment duration was 10.5 months, ranging from 6 to 20 months, with 50% completing treatment within a maximum of 10 months.

## Discussion

The primary objective of this study was to correlate therapeutic interventions categorized into Groups I, II, and III with various patient factors in the context of myofascial pain TMDs. Analysis involved 1,000 medical records, and 348 met the inclusion criteria, representing individuals treated at the Temporomandibular Dysfunction Orofacial Pain Clinic of the Dental Research Center from May 2015 to March 2020.

The observed predominance of female patients in all therapy groups aligns with similar findings by Ratnayake *et al*. (2020) (19), emphasizing the heightened risk of widespread pain in young and middle-aged women, underscoring the need for further investigation into the most effective ways to manage pain in this group. The study also identified prolonged painful symptoms and prior treatments, with 77% experiencing pain for over six months, and 56.7% having undergone prior treatment in therapy Group III. Average treatment durations for Groups I, II, and III were 5.4, 7.5, and 10.5 months, respectively, consistent with prior studies (20). Most myofascial pain diagnoses did not exhibit clinical limitations, suggesting potential diagnostic utility.

While examining patients diagnosed with myofascial pain ( Table 2), 83.4% in Group I, 58.5% in Group II, and 67.3% in Group III exhibited no clinical limitations or symptoms related to TMD. This concurs with Kraus *et al*. (2014), finding 84% of myofascial disorders not restricting mouth opening (21). However, diagnostic utility could be derived from differences in jaw-opening forces (19), aligning with Hong *et al*. (2006), emphasizing effective therapies like manual techniques, physical therapy, and dry needling to eliminate perpetuating factors for pain management (22).

In terms of myofascial TMD pain, prior medication, comorbidity, prior treatment, intensity of the complaint, and diagnosis limitations or without limitations of TMD clinical symptoms were statistically different depending on the type of therapy and were proportionally higher in Group II and Group III therapies (*p* < 0.05). Although there is no consensus in the literature regarding one substance or needle being preferable (23). The likelihood of being in Group II is significantly associated with prior medication (*p*<0.001), prior treatment (*p*=0.002), and clinical diagnosis with limitations (*p*=0.001) when using Group I as a reference. Additionally, a higher chance of being in Group III is linked to clenching as a habit (*p*=0.016), prior medication (*p*=0.003), comorbidity (*p*=0.002), and prior treatment (*p*<0.001).

Despite a high frequency of parafunctional habits, no statistically significant difference was observed for habits and pain duration (*p*>0.05). However, clenching was more prevalent in Groups II (57.0%) and III (63.9%). Habits, present in 100% of patients across groups ( Table 2), are potential factors contributing to TMD.

 Approximately 85.2% of individuals using medication required dry needling and splint therapy, while 46.4% without medication found success with behavioral treatment and thermotherapy (*p*<0.001). For comorbidities, 57.4% with comorbidities needed dry needling and splint therapy, while 76.2% without comorbidities were treated with cognitive-behavioral therapy and thermotherapy (*p*<0.001). This study illustrates the effectiveness of dry needling therapy in relieving pain, especially in Group III, achieving relief within six months (77%). Notably, 45.7% of patients achieved pain relief with Group I therapy in 6 months or less ( Table 2), aligning with Pecos-Martin *et al*. (2015), concluding that dry needling significantly reduces chronic pain (24).

The efficacy of Group I therapy prompts a reevaluation of intraoral devices as the first-choice treatment for myofascial pain. This aligns with previous studies, emphasizing the impact of occlusal splints as additional treatment, impacting psychological aspects (25-27). Another study comparing the efficacy of combination therapy (splint therapy, physiotherapy, manual therapy, and counseling) with physiotherapy, manual therapy, and counseling suggests both approaches in myogenic TMD management (27).

As this study relies on medical record data, it assumes an observational design, limiting control over certain factors and potentially introducing bias. Future well-designed, randomized controlled studies are imperative for a comprehensive assessment of managing myofascial pain.

## Conclusions

Within the limitations of this comparative observational study, the following conclusions can be drawn.

1. Intraoral devices are not the preferred first-line treatment for myofascial pain.

2. Patients without prior treatment, with pain less than 6 months, may be managed with behavioral intervention and thermal intervention in relation to the control of muscular TMD.

3. Patients with prior treatment and pain persisting over 6 months, a combination of behavioral intervention and thermal therapy, along with dry needling, appears effective in optimizing myofascial pain control in the masticatory muscles.

4. Patients with prolonged pain duration, comorbidities, limited mouth opening, a history of medication use, and prior treatments may benefit from the use of a splint in conjunction with other therapies for effective management.

## Figures and Tables

**Table 1:** Sociodemographic characteristics in relation to the adopted therapy type and treatment duration.

	Therapy			p	Treatment		p
	I	II	III		Up to 6 months	6 months or more	
Gender				0,054			0,210
Female	123 (81,5%)	122 (91,0%)	50 (82,0%)		146 (83,0%)	150 (87,7%)
Male	28 (18,5%)	12 (9,0%)	11 (18,0%)		30 (17,0%)	21 (12,3%)	
Age				0,723			0,679
until 39 years	58 (38,4%)	55 (41,0%)	27 (44,3%)		73 (41,7%)	68 (39,5%)	
40 years or more	93 (61,6%)	79 (59,0%)	34 (55,7%)		102 (58,3%)	104 (60,5%)	
Education				0,070			0,616
High school	62 (41,1%)	58 (43,0%)	16 (26,2%)		67 (38,1%)	70 (40,7%)	
Graduate Degree	89 (58,9%)	77 57,0%)	45 (73,8%)		109 (61,9%)	102 (59,3%)	
Occupation				0,574			0,362
None	62 (41,6%)	50 (37,3%)	21 (34,4%)		71 (41,0%)	62 (36,3%)	
Working	87 (58,4%)	84 (62,7%)	40 (65,6%)		102 (59,0%)	109 (63,7%)	

**Table 2:** Behavioral and injury characteristics according to the type of therapy adopted and duration of treatment.

	Therapy			p	Treatment		p
	I	II	III		Up to 6 months	7 months or more	
Habits				0,053			0,051
Clenching	71 (47,0%)	77 (57,0%)	39 (63,9%)		86 (48,9%)	102 (59,3%)	
Others	80 (53,0%)	58 (43,0%)	22 (36,1%)		90 (51,1%)	70 (40,7%)	
Prior medication usage				<0,001			0,001
No	81 (53,6%)	28 (20,7%)	9 (14,8%)		74 (42,0%)	44 (25,6%)	
Yes	70 (46,4%)	107 (79,3% )	52 (85,2%)		102 (58,0%)	128 (74,4%)	
Comorbidity				<0,001			<0,001
Absent	115 (76,2%)	83 (61,5%)	26 (42,6%)		132 (75,0%)	92 (53,5%)	
Exist	36 (23,8%)	52 (38,5%)	35 (57,4%)		44 (25,0%)	80 (46,5%)	
Prior treatment				<0,001			0,014
No	128 (84,8%)	86 (64,7%)	26 (43,3%)		132 (75,9%)	109 (63,7%)	
Yes	23 (15,2%)	47 (35,3%)	34 (56,7%)		42 (24,1%)	62 (36,3%)	
Duration of pain				0,008			<0,001
Until 6 months	69 (45,7%)	50 (37,0%)	14 (23,0%)		87 (49,4%)	46 (26,7%)	
6 months or more	82 (54,3%)	85 (63,0%)	47 (77,0%)		89 (50,6%)	126 (73,3%)	
Diagnosis				<0,001			0,001
No limitations	126 (83,4%)	79 (58,5%)	41 (67,2%)		139 (79,0%)	108 (62,8%)
With limitations	25 (16,6%)	56 (41,5%)	20 (32,8%)		37 (21,0%)	64 (37,2%)
Intensity of the complaint				0,002			0,004
Light	29 (19,2%)	10 (7,4%)	3 (4,9%)		30 (17,0%)	12 (7,0%)	
Moderate or more	122 (80,8%)	125 (92,6%)	58 (95,1%)		146 (83,0%)	160 (93,0%)	

Chi-square.

**Table 3:** Spearman correlation coefficients among quantitative variables.

	Education	Duration of pain	Treatment Durability
Age	-0,198^**^	0,066	0,041
Education		0,054	0,027
Duration of pain			0,317**

*Significant correlation at the 0.001 level (p<0.001).

**Table 4:** Association of participant characteristics with the type of therapy performed (reference category: Therapy I).

	Raw		Adjusted	
	Therapy II	Therapy III	Therapy II	Therapy III
Gender				
Male	Ref.	Ref.		
Female	2,31 (1,13-4,76) p=0,023	1,03 (0,48-2,24) p=0,931	-	-
Age				
40 years or more	Ref.	Ref.		
Up to 39 years	1,12 (0,69-1,80) p=0,650	1,27 (0,70-2,33) p=0,432	-	-
Education				
High school	Ref.	Ref.	Ref.	Ref.
Graduate degree	0,92 (0,58-1,48) p=0,745	1,96 (1,02-3,78) p=0,045	0,82 (0,48-1,40) p=0,466	2,01 (0,94-4,29) p=0,071
Occupation				
None	Ref.	Ref.		
Working	0,84 (0,52-1,35) p=0,461	0,74 (0,40-1,37) p=0,334	-	-
Habits				
Others	Ref.	Ref.	Ref.	Ref.
Clenching	1,50 (0,94-2,39) p=0,091	2,00 (1,08-3,69) p=0,027	1,51 (0,89-2,54) p=0,126	2,38 (1,18-4,81) p=0,016
Pior medication usage				
No	Ref.	Ref.	Ref.	Ref.
Yes	4,42 (2,62-7,47) p<0,001	6,69 (3,08-14,53) p<0,001	3,55 (1,99-6,32) p<0,001	3,74 (1,59-8,81) p=0,003
Comorbidity				
Absent	Ref.	Ref.	Ref.	Ref.
Exist	2,00 (1,20-3,33) p=0,008	4,30 (2,29-8,08) p<0,001	1,30 (0,72-2,34) p=0,380	3,16 (1,52-6,58) p=0,002
Pior treatment				
No	Ref.	Ref.	Ref.	Ref.
Yes	3,04 (1,72-5,37) p<0,001	7,28 (3,70-14,32) p<0,001	2,62 (1,41-4,87) p=0,002	6,36 (3,05-13,27) p<0,001
Duration of pain				
Until 6 months	Ref.	Ref.		
6 months or more	0,70 (0,44-1,12) p=0,139	0,35 (0,18-0,70) p=0,003	-	-
Diagnosis				
No limitations	Ref.	Ref.	Ref.	Ref.
With limitations	3,57 (2,06-6,19) p<0,001	2,46 (1,24-4,88) p=0,010	2,85 (1,56-5,21) p=0,001	1,70 (0,79-3,68) p=0,178
Intensity of the complaint				
Light	Ref.	Ref.		
Moderate or more	0,34 (0,16-0,72) p=0,005	0,22 (0,06-0,74) p=0,015	-	-

**Table 5:** Association of participant characteristics with treatment time (reference category: up to 6 months).

	Raw	Adjusted
Gender		
Male	Ref.	
Female	0,68 (0,37-1,24) p=0,212	-
Age		
40 years or more	Ref.	
until 39 years	1,09 (0,71-1,68) p=0,679	-
Education		
High School	Ref.	
Graduate Degree	0,90 (0,58-1,38) p=0,616	-
Occupation		
None	Ref.	
Working	1,22 (0,79-1,89) p=0,363	-
Habits		
Others	Ref.	Ref.
Clenching	1,53 (0,99-2,33) p=0,051	1,57 (0,99-2,48) p=0,052
Pior medication usage		
No	Ref.	
Yes	2,11 (1,34-3,33) p=0,001	-
Comorbidity		
Absent	Ref.	Ref.
Exist	2,61 (1,66-4,11) p<0,001	2,31 (1,42-3,77) p=0,001
Pior treatment		
No	Ref.	
Yes	1,79 (1,12-2,85) p=0,015	-
Duration of pain		
Until 6 months	Ref.	Ref.
6 months or more	2,68 (1,71-4,19) p<0,001	2,24 (1,39-3,60) p=0,001
Diagnosis		
No limitations	Ref.	Ref.
With limitations	2,23 (1,38-3,59) p=0,001	2,02 (1,21-3,38) p=0,007
Intensity of the complaint		
Light	Ref.	
Moderate or more	2,74 (1,35-5,55) p=0,005	-

**Table 6:** Descriptive analysis of treatment duration according to the therapy adopted.

Therapy	Group 1	Group 2	Group 3
Average (SD*)	5,4(1,9)	7,5(3,0)	10,5(3,1)
Median	5,0	7,0	10,0
Minimum value	2,0	2,0	6,0
Maximum value	12,0	23,0	20,0

## Data Availability

The datasets used and/or analyzed during the current study are available from the corresponding author.
